# Coccidioidomycosis as a Diagnostic Consideration: Expanding the Pulmonary Differential

**DOI:** 10.7759/cureus.110124

**Published:** 2026-06-02

**Authors:** Jane Ma, Patrick H Poquiz

**Affiliations:** 1 Internal Medicine, University of California Los Angeles, Los Angeles, USA

**Keywords:** coccidioides immitis, coccidioidomycosis, coccidiomycosis pneumonia, pulmonary coccidioidomycosis, valley fever

## Abstract

We present a case of a previously healthy 50-year-old man with a two-week history of progressive exertional dyspnea and intermittent sharp pain in the left chest and lower rib area. He then developed cough and low-grade fever, with chest X-ray imaging demonstrating possible left lower lobe consolidation suggestive of bacterial pneumonia. Given the geographic location where the patient lived, with environmental exposure to farming dust, and symptoms that did not improve with antibiotic treatment, alternative diagnoses were considered. The patient was eventually diagnosed with coccidioidomycosis via a positive antibody screen and fungal bronchoalveolar lavage studies that grew *Coccidioides immitis*. He was started on fluconazole with improvement. This case report highlights the clinical presentation, diagnosis, and treatment of this disease, as well as laboratory results noted during subsequent follow-up, which may aid clinicians in the long-term management of these patients.

## Introduction

Coccidioidomycosis, also known as Valley Fever, is a disease primarily thought of as a respiratory illness affecting the pulmonary system but, in some cases, can become disseminated and affect the bones and soft tissues, to name a few sites [1]. It is caused by inhalation of the spores (arthroconidia) of the fungal species Coccidioides (*Coccidioides immitis* or *Coccidioides posadasii*), which are primarily found in the southwestern United States, as well as northern Mexico and Central and South America. There are approximately 150,000 infections per year in the United States, and of those, more than 50% may be asymptomatic [1]. Over the years, there has been an increase in the number of infections, possibly due to increased housing development and construction, travel, and improved testing and detection. Between 2001 and 2011, Los Angeles County had the third-highest average annual number of cases compared to other California counties [2]. More recently, there has been an increasing incidence of symptomatic coccidioidomycosis. While these cases remain largely concentrated in the western United States, the geographic area in which they are found may be expanding, possibly due in part to climate change and travel through endemic areas [3,4]. As such, it is helpful for clinicians to become familiar with possible presenting symptoms and laboratory abnormalities, testing modalities, and treatment options, especially in the setting of persistent or unresolving pneumonia.

## Case presentation

A 50-year-old healthy man was experiencing progressive dyspnea on exertion for a total of two weeks. He reported that he sustained a musculoskeletal injury to his left shoulder while wakeboarding at Castaic Lake, a reservoir just north of Santa Clarita Valley in northwestern Los Angeles County, and since then had been experiencing episodes of sharp pain in the left chest and lower rib area. He initially went to an urgent care center, where he declined an X-ray and was therefore treated conservatively for musculoskeletal pain with a nonsteroidal anti-inflammatory drug (NSAID). Shortly thereafter, he developed a cough and low-grade fever, with temperatures ranging from 99°F to 100.0°F. He also reported postnasal drip and throat irritation, which he felt was causing his cough. He presented again to the urgent care center, where a COVID-19 PCR test was performed and was negative. Subsequently, a chest X-ray suggested possible left lower lobe pneumonia in the presence of a pleural effusion (Figure 1). A complete blood count (CBC) demonstrated a white blood cell (WBC) count of 12,000 at that time (Table 1). He was given a dose of ceftriaxone and subsequently discharged with prescriptions for amoxicillin-clavulanate and azithromycin. He was advised to follow up in 48 hours for reexamination and repeat chest X-ray. Upon returning, he reported a persistent cough and otherwise unchanged symptoms. Repeat laboratory testing showed that his WBC count had increased to 13,600. His initial oral antibiotics were discontinued, and he was started on levofloxacin with instructions to follow up again the next day. Upon his return, he reported that his cough had slightly worsened but otherwise felt that he was breathing normally. Although significant changes would not be expected over such a short interval, repeat chest X-ray showed unchanged left lower lobe airspace disease and pleural effusion. Because the providers were concerned about a possible hemothorax given his recent musculoskeletal injury, he was referred to the emergency department for further evaluation. 

**Table 1:** Laboratory results on presentation to the Emergency Department. WBC: white blood cell; EIA: enzyme immunoassay; HIV: human immunodeficiency virus; IgG: immunoglobulin G; IgM: immunoglobulin M.

Test	Result	Reference Range
WBC	12.0 k/µL	4.5-11.0 k/µL
Eosinophils	5.8%	0.0-3.0%
Aspartate transaminase (AST)	205 U/L	<41 U/L
Alanine transaminase (ALT)	445 U/L	<42 U/L
Alkaline phosphatase	321 U/L	40-130 U/L
Procalcitonin	0.15 ng/mL	0-0.08 ng/mL
Quantiferon Gold	Negative	Negative
Aspergillus antigen EIA	Not detected	<0.50, not detected)
HIV 1 and 2 antibody rapid screen	Non-reactive	Non-reactive
Coccidioides antibody screen	Positive	Negative
Coccidioides IgG	Weakly reactive	Negative
Coccidioides IgM	Reactive	Negative
Coccidioides complement fixation antibody ratio	1:08	<1:2 antibody not detected
Hepatitis A (Hep A) total antibody	Non-reactive	Non-reactive
Hepatitis A (Hep A) IgM	Non-reactive	Non-reactive
Hepatitis C virus (HCV) antibody	Non-reactive	Non-reactive
Hepatitis B (Hep B) core total antibody	Non-reactive	Non-reactive
Hepatitis B (Hep B) core IgM	Non-reactive	Non-reactive
Hep Be antibody	Non-reactive	Non-reactive
Hep Bs antibody	Non-reactive	Non-reactive
Ceruloplasmin	Normal	14-48 mg/dL
Alpha-1 anti-trypsin	236 mg/dL	90-200 mg/dL
Ferritin	615	8-50

**Figure 1:**
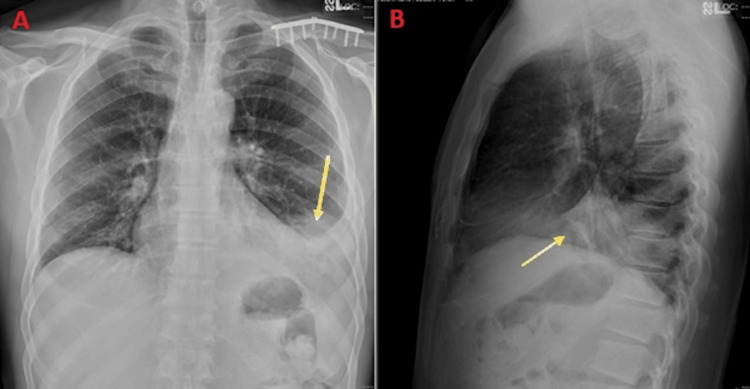
(A) Chest radiograph in PA position and (B) chest radiograph in lateral position obtained from initial Urgent Care visit. (A). A subtle left lower-lobe consolidation is appreciated in the PA (posteroanterior) view and highlighted by the arrow. (B) The lateral view suggests that the consolidation, denoted by the arrow, is adjacent to the left of the spine, behind a faint cardiac silhouette.

On arrival to the emergency department, his temperature was 99.8°F, pulse was normal, respiratory rate was 22 breaths/minute, blood pressure was 146/91 mmHg, and pulse oximetry showed 97% on room air. Physical examination was grossly normal, including the cardiac and pulmonary examinations, apart from mild tenderness to palpation of the left shoulder related to his sporting injury, with full range of motion and no restriction. Laboratory testing again demonstrated an elevated WBC count of 13,600, with differential showing an elevated eosinophil percentage of 5.8%. The comprehensive metabolic panel (CMP) showed hepatocellular injury, with elevated aspartate transaminase and alanine transaminase (AST and ALT) levels of 205 and 445, respectively. He also had an elevated alkaline phosphatase level of 321. His coagulation parameters (PT/INR) were normal, and urinalysis was unremarkable. Computed tomography (CT) of the chest with IV contrast demonstrated dense parenchymal consolidation in the left lower lobe, with evidence of mediastinal lymphadenopathy and multiple small lymph nodes (Figure 2). Given the concern for resistant pneumonia, the decision was made to admit the patient for IV antibiotics and pulmonary and infectious disease consultations.  

**Figure 2:**
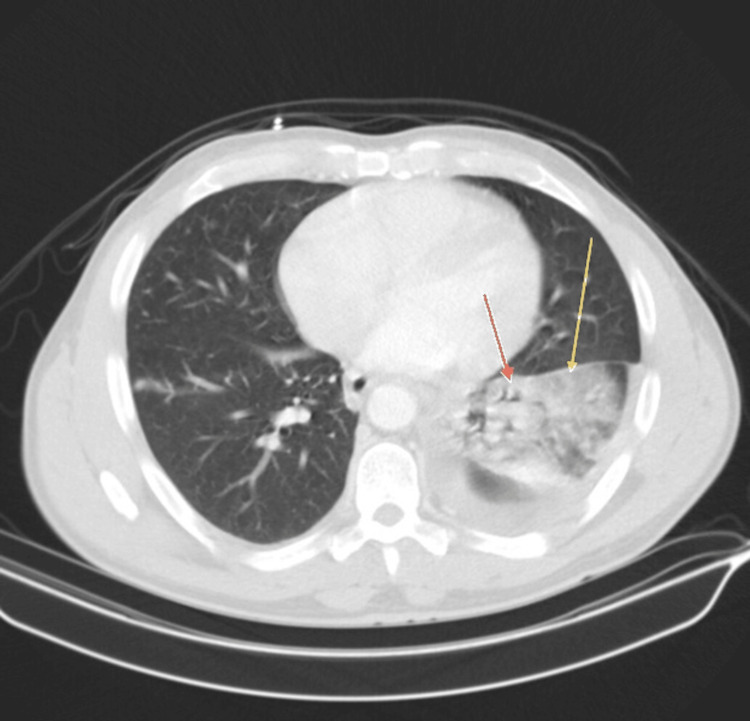
CT (computed tomography) of the chest cross-sectional view. A large, dense parenchymal consolidation is noted at the left lower lobe as highlighted by arrows, with layering left pleural effusion.  Air bronchograms are also noted within the consolidation.

During his hospitalization on the wards, the patient offered more information: several months prior, he had been working on farming land near his house in Castaic, California. During that time, he used a tractor for planting seeds, which agitated the dirt and dust. He did not wear a mask at that time. Given the concern that he may have developed possible pulmonary coccidioidomycosis, the treatment team decided to pursue diagnostics and treatment for this. On admission, he was started on IV piperacillin-tazobactam, but given that he had a relatively low procalcitonin level of 0.15 ng/mL, this was discontinued, and he was started on empiric fluconazole at 600 mg IV q24 hours, given the concern for disseminated disease. Given the concern for a persistent pulmonary infiltrate with possible fungal exposure, diagnostic bronchoscopy with bronchoalveolar lavage (BAL) was also performed in the affected left lower lobe, and other than mild secretions, there were no endobronchial lesions noted. Serum studies that were also obtained included QuantiFERON-TB Gold for tuberculosis, which was negative; Aspergillus antigen EIA, which was negative; HIV-1 and HIV-2 antibody rapid screening, which was nonreactive; and Coccidioides studies, including an antibody screen, which was positive. Additionally, the IgG antibody level was weakly reactive, the IgM antibody was reactive, and the complement fixation antibody titer was 1:8. With regard to his BAL studies, bacterial culture showed only normal respiratory flora, acid-fast stain and mycobacterial culture were negative, but fungal culture was positive for Coccidioides immitis. Prior to his discharge from the hospital, his AST and ALT started to downtrend to 77 and 153, respectively, but his eosinophilia remained persistently high at 12.3%. Given his stability after diagnostic workup, he was ultimately discharged home from the hospital on oral fluconazole 600 mg daily with instructions to follow up with infectious diseases as well as his primary care physician. He was provided antifungal treatment for a total of six months. It is worth noting that his primary care doctor did perform follow-up testing to exclude alternative causes of hepatocellular injury, for which a hepatitis panel including Hep A total Ab, Hep A IgM, HCV Ab, Hep B core total Ab, Hep B core IgM, HBe Ab, and HBs Ab was unremarkable. Ceruloplasmin was normal, and alpha-1 antitrypsin and ferritin were only mildly elevated at 236 and 615, respectively. Fortunately, his complement fixation serum levels improved with treatment after five months, with results from that time and onward showing <1:2 ratio.

## Discussion

With respect to presentation and the spectrum of disease, coccidioidomycosis can present in various ways. As noted above, some patients may be asymptomatic, but many present with symptoms such as fever, cough, chills, diaphoresis, weight loss, and bony or muscle pain. Pulmonary involvement can manifest as mild to moderate disease or as more severe and progressive disease, including complications such as pleural effusion, nodules, or cavitary and fibrocavitary disease. Disseminated disease is less common and can include development of osteomyelitis, synovitis, soft tissue infections, cutaneous manifestations, peritonitis, lymphadenopathy, and meningitis. In the case of the patient above, in addition to pulmonary manifestations, he was noted to have liver function test (LFT) abnormalities that were initially thought to be from coccidioidomycosis. Liver injury can be observed in disseminated coccidioidomycosis but is less apparent in immunocompetent patients [5]. Often, the LFT abnormalities noted include moderately elevated AST and ALT levels [6]. Hyperbilirubinemia is rare in immunocompetent persons. Pulmonary and hepatic involvement in coccidioidomycosis is thought to result in milder disease clinically, in contrast to involvement of other areas such as the central nervous system (CNS). In immunocompromised persons, use of corticosteroids, biologics associated with gastrointestinal or dermatologic conditions, TNF-α inhibitors, or the Janus kinase inhibitor ruxolitinib is associated with an increased risk of symptomatic, recurrent, or extrathoracic dissemination [3].

Coccidioidomycosis is diagnosed clinically as well as through laboratory, microbiological, serological, and radiographic workup, as delineated in the above patient's case. Laboratory findings that can be found in patients with coccidioidomycosis include eosinophilia, polyclonal hyperglobulinemia, and hypoalbuminemia. C-reactive protein and erythrocyte sedimentation rate levels are often elevated. White blood cell count can vary. Procalcitonin is usually normal. Diagnosis can be made with serum or cerebrospinal fluid anti-coccidioidal antibodies. False-negative serologic results are common early in the disease course. Enzyme immunoassay (EIA) testing for IgM and IgG antibodies is sensitive but less specific, and false-positive IgM results can be seen. Thus, confirmation should be obtained via complement fixation testing. Complement fixation (CF) titers are ordered to measure IgG antibodies; in general, lower titers of <1:32 are seen in uncomplicated disease, whereas higher titers of >1:32 are seen in more complicated disease [7]. The CF titer is associated with disease prognosis and is frequently followed to monitor response to treatment, as demonstrated in our patient, who was fortunate to have a titer of <1:2 after five months of treatment [3]. Serum beta-D-glucan is a commonly used laboratory test in the inpatient setting that can aid in the investigation of a possible fungal infection, and it can be elevated in disseminated coccidioidomycosis, but it does not always correlate with serum Coccidioides CF titers [8]. Ultimately, a diagnosis based on culture or histopathology results is definitive [1]. Additional workup, including chest X-ray, is typically performed and may reveal an infiltrate, hilar adenopathy, or pleural effusion [8]. Diagnostic imaging should also be performed for further evaluation of areas of concern, including CT or MRI if there is concern for bony involvement.

Available treatment options include medications from the polyene and azole classes, with amphotericin B and ketoconazole being the first FDA-approved agents; however, both are associated with significant side effects. Treatment is typically initiated with an azole, usually fluconazole, given its more favorable side effect profile, low cost, and bioavailability, and thus it was chosen for our patient [9,10]. Treatment failure often results in transition to another azole. Azoles can carry a risk of hepatotoxicity, QT prolongation (for most azoles), and other side effects. Amphotericin B is used if the above therapies are not effective or in cases of severe, progressive disease [7]. Notable side effects of amphotericin B include nephrotoxicity, hypokalemia, myalgias, and anemia. Newer treatment options are being explored and are in development [3]. For pulmonary disease, treatment duration can vary from three to 12 months, with treatment discontinued when symptoms resolve and laboratory and radiographic evidence of improvement is noted. As noted for the patient above, there was improvement in CF levels after five months. For disseminated disease, treatment duration can extend up to three years or more until clinical and radiographic stability.

## Conclusions

One is taught in medical training to identify certain regions as endemic for coccidioidomycosis, but one can mistakenly miss the diagnosis by assuming that the disease is only apparent in desert and dusty climates, which include several areas in Los Angeles. It is important to recognize that the diagnosis still needs to be included in the differential when considering presentations outside of endemic areas or areas that do not fit the stereotype. Although at a lower incidence rate, non-endemic U.S. states in total account for up to one-third of reported cases, many times associated with patients who have traveled to endemic areas but do not actually reside in those areas. In those instances, obtaining a travel history would be imperative; however, in our case presentation, environmental exposure history was critical in the diagnostic process. Improving diagnostic awareness in the United States would significantly benefit the public health response, as there is a large gap and discrepancy between reported and estimated cases, which in turn can reduce misdiagnoses and inappropriate treatments for more common pulmonary infections such as community-acquired pneumonia. Early identification and diagnosis serve several benefits, including reduction of mortality in severely immunocompromised patients, prevention of progression and reactivation in latent infections, and improvement of epidemiologic data to strengthen overall resource allocation for appropriate treatment.

This case highlights the importance of recognizing potential infection with coccidioidomycosis and including this disease in the differential diagnosis, given its increasing prevalence and impact on quality of life. From a clinical practice standpoint, coccidioidomycosis should be considered in instances where pneumonia persists despite antibiotic treatment, eosinophilia is present, or the history is suggestive of environmental or travel exposure. It further underscores the importance of access to primary and specialty health care, as in some cases, follow-up is needed for months or years to determine response to treatment. Timely diagnosis of coccidioidomycosis would aid in minimizing unnecessary antibiotic use and avoiding delays in treatment.
